# Screening, diagnosis and genetic study of breast cancer patients in Pakistan

**DOI:** 10.12669/pjms.36.2.1059

**Published:** 2020

**Authors:** Ayesha Isani Majeed, Asmat Ullah, Muniba Jadoon, Wasim Ahmad, Sheikh Riazuddin

**Affiliations:** 1 Ayesha Isani Majeed Department of Radiology, Pakistan Institute of Medical Sciences, Islamabad, Pakistan. Health Services Academy, Islamabad, Pakistan; 2 Asmat Ullah, Department of Molecular Biology, Shaheed Zulfiqar Ali Bhutto Medical University, Islamabad, Pakistan. Department of Biochemistry, Faculty of Biological Sciences, Quaid-i-Azam University, Islamabad, Pakistan; 3 Muniba Jadoon, Department of Molecular Biology, Shaheed Zulfiqar Ali Bhutto Medical University, Islamabad, Pakistan; 4 Wasim Ahmad, Department of Biochemistry, Faculty of Biological Sciences, Quaid-i-Azam University, Islamabad, Pakistan; 5 Sheikh Riazuddin, Department of Molecular Biology, Shaheed Zulfiqar Ali Bhutto Medical University, Islamabad, Pakistan

**Keywords:** Breast Cancer, Sanger Sequencing, *BRCA1* Variants

## Abstract

**Objective:**

To determine the role of variants in *BRCA1* gene in breast cancer development, women of Pakistani origin, diagnosed with breast cancer, were screened for variants in the *BRCA1*.

**Methods:**

The present study involved screening of 5000 women for breast cancer. 302 women were diagnosed with breast cancer. Using Sanger sequencing, DNA extracted from peripheral blood of 100 patients was screened for disease causing variants in the *BRCA1*.

**Results:**

Analysis of sequenced data revealed two frame shift (Gly312Trpfs*8, Ala322Glyfs*4), six missense (p.Glu362Lys, p.Lys651Arg, p.Asp693Asn, p.Pro871Leu, p.Glu1134Lys, p.Lys1183Arg), four synonymous (p.Thr327Thr, p.Ser694Ser, p.His771His, p.Gln1135Gln), and two intronic variants (g.75407T>C, g.75401_75401delT) in the patients.

**Conclusion:**

The present investigation showed that variations in *BRCA1* made substantial contribution in causing hereditary/early-onset breast cancer in Pakistani women.

## INTRODUCTION

Breast cancer is designated as one of the major life threatening malignancies throughout the world. As reported by World Health Organization (WHO, 2018), the worldwide incidence is on the rise and attributed to factors like increased life expectancy, increased urbanization and adopted lifestyle. In another report issued by World Cancer Research Foundation, nearly 1.7 million new cases were diagnosed in 2012 worldwide making it second most common cancer overall (www.wcrf.org/int/cancer-facts-figures/data-specific-cancers/breast-cancer-statistics). In Pakistan a comprehensive analysis revealed that breast cancer is the most prevalent, frequently diagnosed and top most common cause of cancer death among the female population.^1^ These authors reported 119,710 cases of breast cancer in the last five years.

In the last few years, several published studies have reported several factors contributing towards high incidence of breast cancer. This included high penetrance of inherited cases resulting from genetic mutations.^2^ To date, two major susceptibility genes, *BRCA1* and *BRCA2*, involved in development of breast cancer, have been identified. Genomic rearrangements within these genes are reported to have sound association with breast cancer.^3^ *BRCA1* is a tumor suppressor gene, located on chromosome 17q21, and composed of 22 coding and two non-coding exons.^4^ It has a transcript of 7.8kb which encodes 1863 amino acids nuclear protein, and play highly pivotal role in transcription and DNA damage repair mechanism.^5^

It has been reported that mutational changes in the *BRCA1* confer 80% life time risk of breast cancer and 40% of ovarian cancer.^6^ Although, over 500 alterations have been documented for *BRCA1*, nevertheless the mutational spectrum for this gene has not been characterized entirely. This is due to the fact that these mutational changes revealed varying distribution among different ethnicities and across diverse geographic regions.^7^,^8^

Pakistan is a geographically dynamic country and home to population with varying ethnicities and geographical linkages. Therefore, there is a need to conduct molecular analyses among the breast cancer patients across different regions of the country and contribute towards defining statistics at the national level. Also, various factors like genetics, life style, obesity, exposure to ionizing radiations have been associated with an increase in cancer. Therefore early detection and breast cancer awareness are the key towards longevity and survival against the disease.^9^ Previously, genetic alterations in the *BRCA1* in Pakistani breast cancer patients have been reported only in two different studies.^6^,^10^ The present study was conducted at the Federal Capital Territory so as to comprehend the mutational changes in *BRCA1* among the breast cancer patients of the region.

## METHODS

In total, 5000 females were screened for breast cancer from October 2015 to October 2018 at Federal Breast Cancer Screening Centre, Pakistan Institute of Medical Sciences, Islamabad. The study was approved by Ethical Review Board of Shaheed Zulfiqar Ali Bhutto Medical University, Quaid-i-Azam University Islamabad (Ref. No. IRB-QAU-183 dated January 22, 2019). Patients recruited in the study had a diagnosis of primary invasive breast cancer. They were selected irrespective of the patients’ age or breast cancer stage at the time of diagnosis. Both the prevalent as well as incident cases were included in the study. Clinical, histopathological and risk factor data such as age, marital status, consanguinity, parity, family history of breast cancer and ethnic group were collected for all the participants using a questionnaire. The patients were screened and diagnosed on the basis of mammography, ultrasound, followed by fine needle aspiration and tru-cut core biopsy. The specimen was collected in Formalin in a container labeled with patient name and surgical number. The equipment used was Toshiba mammography machine and histologic computed tomography machine with contrast enhanced tomography facilities. Women having age more than 40 were screened using mammography test. Mammography cannot detect tumor in dense breast tissue, while younger women are more likely to have dense breast tissue, therefor for those individuals, ultrasound was recommended. For those with BIRADS-IV and BIRADS-V lesions biopsies were undertaken, whereas for those with BIRADS-III lesions, the clinician recommended those patients with a family history for biopsy. A cut off of BIRAD-IV or above was considered as positive case of breast cancer, which was confirmed by histopathology. Ultrasound guided biopsy was done for diagnostic purposes using stereotactic biopsy facilities.

For genetic study, first one hundred cases with breast cancer were selected irrespective of their age, stage of cancer or ethnic group from a consecutive series of 302 patients with a first diagnosis of breast. Peripheral blood samples of the patients were collected in 5mL EDTA coated tubes and stored at 4°C before further use. All study participants signed informed written consent.

### Inclusion criteria

1. Women with early onset breast cancer with or without family history of breast cancer.
2. Women with breast cancer having family history of breast or ovarian cancer irrespective of age at diagnosis.

### Extraction of DNA

Genomic DNA was extracted from 5ml of the blood samples using genomic DNA extraction kit (PureLink^™^ Genomic DNA Mini Kit, Thermo Scientific USA) according to manufacturer’s instruction. Following extraction, the quantification of the genomic DNA was carried out by measuring the optical density at 260 nm using spectrophotometer. The samples were diluted to 40 ng/ µL to 50 ng/µL for further use.

### Genetic Investigation

DNA samples were screened for mutations in the coding regions of *BRCA1* using PCR amplification followed by Sanger sequencing. The pairs of oligonucleotide primers were designed from intronic sequences of the gene using Primer3 software (bioinfo.ut.ee/primer3-0.4.0) (Supplementary Table-I).

**Table-I:** A table showing demographics of studied population.

Ethnic Group	Percentage
Punjabi	73%
Pashtuns	15%
Kashmiries	6%
Others (Balochi, Sindhi, Muhajir)	6%

The PCR reaction volume was 25 µL containing 40 ng genomic DNA, 20 pmol of each primer, 200 mmol of each deoxyribonucleotide triphosphate, 1U of Taq DNA polymerase and 2.5 µL of the buffer. All the chemicals were procured from Fermentas Inc (York, UK). The amplification conditions used were the same as described previously by Ullah *et al*.^11^ The amplified products were resolved on 2% agarose gel, stained with ethidium bromide and viewed using transilluminator. The products were purified and sequenced using Big Dye terminator cycle sequencing kit v.3.1 (Applied Biosystems, USA). The samples were aligned and analyzed for sequence variation using software BIOEDIT (version 6.0.7). Pathogenicity score for the identified variant was calculated using mutation Taster (http://www.mutationtaster.org/).

## RESULTS

### General Characteristics of Patients

**Fig.1:**
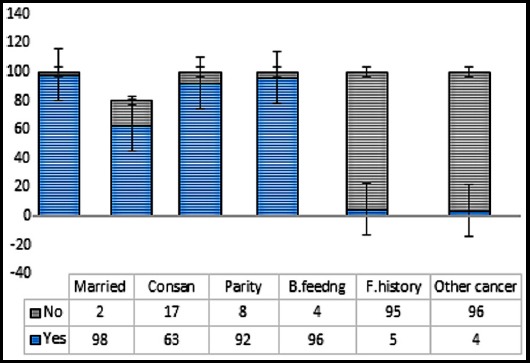
General characteristics of the breast cancer patients recruited for screening BRCA1 for disease causing variants.

**Abbreviations:** Consan= consanguinity; B.feeding= Breast feeding; F.history= Family history

In total, 5000 women were screened at Federal Breast Cancer Screening Centre, Pakistan Institute of Medical Sciences, Islamabad during the period of three years (October 2015 to 2018). Out of which 302 women were diagnosed with breast cancer. Cancer was diagnosed base upon radiological and histopathology findings. A total of one hundred patients, all females, diagnosed with breast cancer were recruited in the study irrespective of their age or stage at the time of diagnosis. Analysis of the general characteristics showed that the study group was dominated by patients of Punjabi origin, which was further dominated by patients from Islamabad Capital Territory and Upper Potohar region of the country (Table-I). It was followed by patients of Kashmiri and Pashtun origin. The median age of the patients was 46 years with majority between 36 and 45 years of age. As shown in the Fig-1, 98% of the cases were married, of which 63% were consanguineous marriages. The average parity was four with positive breast feeding history among 96% of the cases. Family history of breast cancer was noted among only 5% while other cancer type was positive among 4% of the cases (Fig.1).

### Genetic Investigation

Blood samples collected from 100 diagnosed breast cancer patients were screened for mutations assuming that these alterations could play a significant role in the pathogenesis of breast cancer. In total, two frame shift (p.Gly312Trpfs*8, p.Ala322Glyfs*4), six missense (p.Glu362Lys, p.Lys651Arg, p.Asp693Asn, p.Pro871Leu, p.Glu1134Lys, p.Lys1183Arg), four synonymous (p.Thr327Thr, p.Ser694Ser, p.His771His, p.Gln1135Gln), and two intronic variants (g.75407T>C, g.75401_75401delT) were detected in the *BRCA1* among 79 of the total 100 patients (summarized in Table-II).

**Table-II:** List of mutations found in the gene BRCA1 in the present study.

Type of mutation	Nucleotide change	Protein	Recurrent/Novel
Frameshift	c.932_933insC	Gly312Trpfs*8	Novel
Frameshift	c.964_965insG	Ala322Glyfs*4	Novel
Missense	c.1084G>A	Glu362Lys	Novel
Missense	c.1952A>G	Lys651Arg	Novel
Missense	c.2077G>A	Asp693Asn	Recurrent
Missense	c.2612C>T	Pro871Leu	Recurrent
Missense	c.3400G>A	Glu1134Lys	Novel
Missense	c.3548A>G	Lys1183Arg	Recurrent
Synonymous	c.981A>G	p.Thr327Thr	Novel
Synonymous	c.2082C>T	p.Ser694Ser	Novel
Synonymous	C.2311T>C	p.His771His	Novel
Synonymous	c.3405G>A	p.Gln1135Gln	Novel
Intron Variant	g.75407T>C		Novel
Intron Variant	g.75401_75401delT		Novel

Following mutational analysis, clinical-pathological features were analyzed in samples with or without *BRCA1* alterations. Results showed that women found positive for *BRCA1* mutations were younger (36 years) and expressed higher rate of lymph node involvement as compared to women without alterations. Similarly, *BRCA1* group was dominated by cases expressing bilaterally affected breast tissues with lower expression of estrogen (ER) and progesterone (PgR) receptors as compared to non-*BRCA1* cases.

## DISCUSSION

All the patients recruited in the study were selected at Federal Breast Cancer Screening Centre. This dedicated breast cancer screening facility is situated at the heart of the Federal Capital and is visited by the patients from all provinces of Pakistan. Thus the data collected from patients visiting the center could help to delineate the mutational spectrum of the population of the region and contribute towards designing improvised awareness and control strategies. The data analysis showed that breast cancer patients presenting *BRCA1* mutations were mostly younger (≤ 45 years of age), married, and had positive history of breast feeding and child bearing. The median age at the time of diagnosis was found to be 48.9 years.

Four different types of mutations were detected in DNA extracted from multiple unrelated patients (Table-II). These included frameshift, missense, synonymous and intron variations. The two frameshift variants (p.Gly312Trpfs*8, p.Ala322Glyfs*4) are predicted to result in loss of function of the BRCA1 protein either through nonsense-mediated mRNA decay or resulting in production of truncated protein, subsequently affecting DNA-repair mechanism. Three of six missense mutations (p.Asp693Asn, p.Pro871Leu, p.Lys1183Arg) have been described previously in other populations^12^-^14^ but not in Pakistani population. Three other missense variants (p.Glu362Lys, p.Lys651Arg, p.Glu1134Lys) were novel and not reported previously in any population. These three variants are located between the nuclear localization signals and the C-terminus of BRCA1 which is involved in the interaction of BRCA1 with a number of proteins that function in the DNA repair process and the cell cycle checkpoint control.^15^ The two intronic variants (g.75407T>C, g.75401_75401delT) identified in present study are predicted to affect the gene expression. It is highly likely that these nucleotides in the intronic regions are part of regulatory sequences of the gene affecting its expression level.

Comparison of clinical pathological features observed among women with and without alterations in the *BRCA1* revealed the former were found to have high cyto-histological tumor grade, showed nodal involvement and expressed low levels of progesterone and estrogen receptors. These findings are consistent with studies previously reported from Pakistan and other parts of the world. Rashid *et al.^1^*6 have reported that Pakistani women are usually diagnosed with breast cancer below 40 years of age, often present high tumor grade and show poor rate of lymph node involvement. Similarly, Leide and colleagues reported median age of diagnosis as 41 years.^17^ A hospital based study on breast cancer patients from Apulia, Italy supports our findings as it reported ductal invasive type, higher cytohistological tumor grade, negative ER and PgR status in the *BRCA1* associated carcinomas.^14^ A possibility cannot be excluded that patients found negative for variants in the *BRCA1* have variations in the *BRCA2*.

Pakistan is a country that has faced massive migration of population and is hence composed of varying ethnic groups.^18^ The findings of the present study present varying mutational spectrum across different ethnic groups. Although, the study recruited relatively small number of patients and presents limited data in terms of age, ethnic group, etc., nevertheless it contributes to the studies conducted earlier by adding up to the different mutational changes described for the Pakistani population so far. This study although provides an insight into mutational spectrum of the patients in the region nevertheless it offers several limitations. The moderate sample size limited the statistical analysis. Henceforth, further population based case control studies with relatively larger sample size are suggested.

## CONCLUSION

Genetic factors in the form of *BRCA1* mutations are significant contributors to the high prevalence of breast cancer among Pakistani population. Therefore genetic testing for *BRCA1* mutations along with *BRCA2* is highly crucial in order to streamline early detection and hence reduce mortality of breast cancer.

### Authors’ Contribution:

**AIM, AU** recruited samples and collected/compiled clinical information with help of **MJ, AIM,** **AU, MJ,** performed genetic studies, and analyzed data alongside **WA,** Manuscript writing and revision: **WA, SR.** Study supervision and coordination:

All the authors critically revised the manuscript and contributed to the discussion. The final version of the paper was read and approved by all authors.
